# Supplementary material for: MetaRibo-Seq measures translation in microbiomes
Source: Nat Commun. 2020 Jun 29;11:3268. doi: 10.1038/s41467-020-17081-z (PMC7324362; doi:10.1038/s41467-020-17081-z)
Supplement: Supplementary file 10 — Supplementary Data 7 [file 41467_2020_17081_MOESM10_ESM.zip › File2/Confidence_High_Taxonomy/112440_out.krona.html]

Javascript must be enabled to view this page.

members
magnitude
magnitudeUnassigned
count
unassigned
taxon
rank

112440\_out

3

superkingdom
2
3

3
phylum
201174

3
class
84998

order
1643822
3

3
1643826
family

3
644652
genus

471189
species
3

SRS015782\_contig\_number\_contig-100\_2666.217134SRS053214\_contig\_number\_contig-100\_1123.97341SRS147346\_contig\_number\_53915
